# An Observational Study to Evaluate the Association between Intestinal Permeability, Leaky Gut Related Markers, and Metabolic Health in Healthy Adults

**DOI:** 10.3390/healthcare9111583

**Published:** 2021-11-19

**Authors:** Hiroyuki Hoshiko, Gertrude G. Zeinstra, Kaatje Lenaerts, Els Oosterink, Renata M. C. Ariens, Jurriaan J. Mes, Nicole J. W. de Wit

**Affiliations:** 1 HE Center, Suntory MONOZUKURI Expert Limited, Kyoto 619-0284, Japan; 2 Wageningen Food and Biobased Research, Wageningen University & Research, 6708 WG Wageningen, The Netherlands; gertrude.zeinstra@wur.nl (G.G.Z.); els.oosterink@wur.nl (E.O.); renata.ariens@wur.nl (R.M.C.A.); jurriaan.mes@wur.nl (J.J.M.); nicole.dewit@wur.nl (N.J.W.d.W.); 3 Department of Surgery, NUTRIM School of Nutrition and Translational Research in Metabolism, Maastricht University, 6200 MD Maastricht, The Netherlands; kaatje.lenaerts@maastrichtuniversity.nl

**Keywords:** metabolic, intestinal permeability, multi-sugar, leaky gut

## Abstract

We explored whether metabolic health is linked to intestinal permeability, using a multi-sugar (MS) permeability test, and whether intestinal permeability is correlated with the leaky gut-related markers (LGM) zonulin, LBP, and sCD14. Metabolically healthy (*n* = 15) and unhealthy subjects (*n* = 15) were recruited based on waist circumference, fasting glucose, and high-density lipoprotein cholesterol levels. Participants underwent an MS permeability test that assessed site-specific permeabilities of the gastroduodenum and small and large intestines. The test was performed with/without an acetylsalicylic acid challenge to measure and correlate the gut permeability, LGM, and metabolic health. At baseline, metabolic health showed no correlation with gut permeability. Significant correlations were found between the metabolic health parameters and LGM. In the acetylsalicylic acid challenged MS permeability test, low-density lipoprotein cholesterol was correlated with the sucralose/erythritol ratio, reflecting the whole intestinal permeability. Correlations between most metabolic health parameters and LGM during the acetylsalicylic acid challenge were less pronounced than at baseline. In both MS permeability tests, no significant correlations were found between LGM (plasma and serum) and gut permeability. Thus, correlations between LGM and metabolic health might not be linked with paracellular gut permeability. Transcellular translocation and/or lipoprotein-related transportation is a more likely mechanism underlying the association between LGM and metabolic health.

## 1. Introduction

Chronic inflammation is an underlying cause of the development and progression of obesity and metabolic syndrome [1]. It refers to a prolonged inflammatory response that may lead to cell damage and may affect physiological homeostasis [2]. Inflammation related to obesity affects the expression of adiponectin and tumor necrosis factor (TNF)-α in adipose tissue, which leads to the secretion of other proinflammatory cytokines [3]. Levels of the proinflammatory cytokines are increased in the plasma of obese individuals with metabolic syndrome and type 2 diabetes mellitus (T2DM) [4]. Therefore, the suppression of chronic inflammation is an important target for preventing the development and progression of lifestyle-related diseases. However, the causes and mechanisms of chronic inflammation remain unclear. The gastrointestinal tract is constantly exposed to dietary allergens and commensal bacteria [5]. Thus, increased intestinal permeability may be a risk factor for the translocation of potentially harmful compounds. ‘Leaky gut’ is a term reflecting increased intestinal permeability and may be responsible for a large variety of health issues [6]. It has been reported that increased intestinal permeability is associated with gastrointestinal disorders, such as irritable bowel syndrome (IBS) [7], inflammatory bowel disease (IBD) [8], and celiac disease [9]. Another recent study showed that increased intestinal permeability is associated with visceral adiposity and liver fat accumulation [10], which are both closely related to other metabolic disorders, such as insulin resistance and elevated low-density lipoprotein–cholesterol levels. In our previous study, we explored leaky gut-related markers (LGM) that can be measured in plasma or serum and are correlated with an unhealthy metabolic profile in the Dutch population; the most relevant LGM was zonulin [11]. A previous study also reported increased circulatory levels of zonulin and lipopolysaccharides (LPS) in patients with T2DM [12]. Recent studies have shown that circulating levels of lipopolysaccharide-binding protein (LBP) and sCD14 are correlated with inflammatory and metabolic markers, such as cholesterol and/or triglycerides [11,13]. These findings indicate that a ‘leaky gut’ might have a direct or indirect role in the development of metabolic disorders related to metabolic syndrome [14]. There is growing evidence that suggests that obesity and/or metabolic health are related to intestinal permeability, as assessed by a dual sugar test, which mainly reflects paracellular permeability [10,15,16]. In particular, a link between visceral fat and increased permeability in the lower gastrointestinal tract has been found in human studies. For instance, Farhadi et al. [17] concluded that increased colon permeability in nonalcoholic steatohepatitis (NASH) patients likely resulted in increased serum endotoxin levels and contributed towards health problems.

We performed a trial to explore whether metabolic health parameters and LGM could be linked to intestinal permeability, as assessed by a sugar test. For this, we used the multi-sugar (MS) permeability test instead of a dual-sugar test, as the MS permeability test provides more accurate, site-specific information on gastroduodenal, small, and large intestinal paracellular permeability [18]. The MS permeability test was performed with and without an acetylsalicylic acid challenge, as it was previously reported by Farhadi et al. [17] that an acetylsalicylic acid challenge was needed to more accurately detect significant differences in the gut permeability between people with different metabolic health statuses.

## 2. Materials and Methods

### 2.1. Subjects

**Figure 1:**
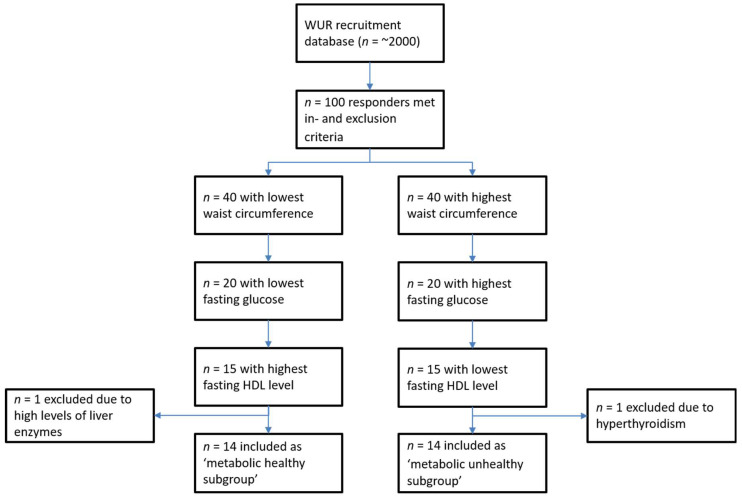
Overview of the selection procedure.

The inclusion criteria for this study were as follows: apparently healthy and age 20–70 years. The participants of this study were recruited from the surrounding areas of Wageningen, The Netherlands. The exclusion criteria for this study were as follows: (1) a history of gastrointestinal disorders (stomach ulcer, ulcerative colitis, Crohn’s disease, and celiac disease); (2) a history of gastrointestinal surgery; (3) a history of liver dysfunction (cirrhosis and hepatitis); (4) diabetes mellitus; (5) a history of acute coronary syndrome; (6) heart failure; (7) kidney dysfunction (eGFR < 60 mL/min); (8) thromboembolic disorders; (9) an intake of medications known to change the inflammatory status (i.e., proton pump inhibitors, antibiotics, and anti-inflammatory medication (including nonsteroidal anti-inflammatory drugs (NSAIDs)); (10) hypersensitivity to NSAIDs or the sugars in the MS mix; (11) pregnancy (self-reported, not tested within the study); (12) age below 20 or over 70 years; (13) alcohol intake ≥40 g/day (≥3 to 4 glasses of beer/wine per day); (14) drug abuse; (15) current smokers; and (16) participation in other clinical trials in the past month. The total number of 30 subjects was divided into two groups (*n* = 15 each) differing in metabolic health (metabolic healthier and metabolic unhealthier) based on the waist circumference, fasting glucose, and high-density lipoprotein (HDL)–cholesterol level. An overview of the selection procedure is presented in Figure 1. 

### 2.2. Ethical Considerations

This study was approved by the Medical Ethical Committee of Wageningen University. Furthermore, the study was conducted according to the principles of the Declaration of Helsinki, in accordance with the Medical Research Involving Human Subjects Act (WMO), and registered in the Netherlands Trial Register (NL4059). All subjects provided written informed consent prior to participating in the study.

### 2.3. Study Design

**Figure 2:**
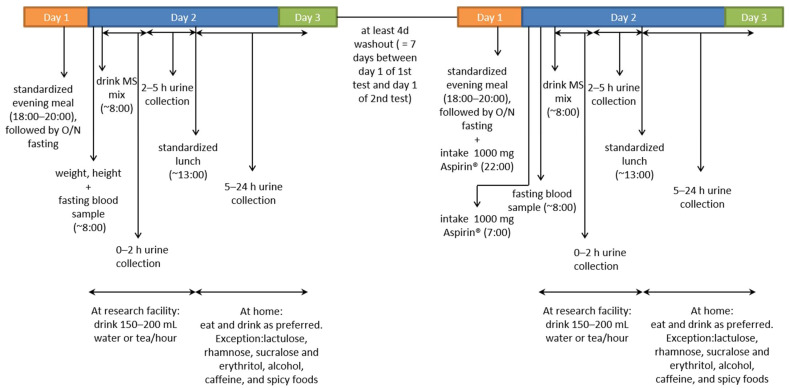
Schematic overview of the study design.

This was an observational study in which gut permeability was assessed using a multi-sugar (MS) permeability test. The MS permeability test was performed with and without an acetylsalicylic acid challenge; therefore, each subject visited the research facility twice. On the day of the MS permeability test, weight and height were first measured, and a fasting blood sample was then collected. Subsequently, the subjects consumed 200 mL MS mix solution containing 1 g sucrose (van Gilse, Dinteloord, The Netherlands), 1 g lactulose (Centrafarm, Etten-Leur, The Netherlands), 0.5 g L-rhamnose (Danisco, Copenhagen, Denmark), 1 g sucralose (Brenntag, Sittard, The Netherlands), and 1 g erythritol (Now Foods, Bloomingdale, IL, USA). Urine was collected to measure the gut permeability, as described below. Five hours after drinking the MS mix, subjects received a standardized lunch and continued urine collection at home for 19 h, where they were allowed to eat normally (with restrictions for consuming lactulose, rhamnose, sucralose, erythritol, alcohol, caffeine (including tea), and spicy foods). During the second visit, at least four days later, the same procedures were repeated, except that the subjects consumed 1000 mg acetylsalicylic acid (Aspirin^®^, Bayer AG, Leverkusen, Germany) in the evening and 1000 mg in the morning prior to the MS permeability test, as outlined in detail below. A schematic overview of the study design is presented in Figure 2.

### 2.4. MS Gut Permeability Test

The MS gut permeability test was performed as described by van Wijck et al. [18]. All sugars were food-grade, safe for oral consumption. The sugar probes were 95–99.9% pure. After consumption of the MS mix, urine was collected in three fractions (fraction 1: 0–2 h, fraction 2: 2–5 h, and fraction 3: 5–24 h) to enable accurate localization of the permeability in the gut, which was determined by the urinary excretion (%) of sucrose and the sucrose/rhamnose ratio in fraction 1. Permeability of the small intestinal was estimated by measuring the urinary excretion (%) of lactulose and the lactulose/rhamnose ratio in fractions 1 (proximal small intestine) and 2 (distal small intestine). The colonic permeability was estimated by measuring the urinary excretion (%) of sucralose and the sucralose/erythritol ratio in fraction 3, and the excretion (%) of sucralose and the sucralose/erythritol ratio in all three fractions (fraction 1–3: 0–24 h) reflected whole intestinal permeability. The MS permeability test was conducted with and without (unchallenged) an acetylsalicylic acid challenge, with a washout period of at least four days between the tests (the test was conducted in this order for all subjects). The acetylsalicylic acid challenge involved pretreatment with 2000 mg acetylsalicylic acid: acetylsalicylic acid was provided as Aspirin^®^ 500 mg tablets from Bayer. It is reported to exacerbate gut permeability, thereby facilitating the detection of differences in gut permeability [19].

Sugars were detected in the urine fractions by isocratic ion-exchange high-performance liquid chromatography (HPLC) (Model PU-1980 pump, Jasco Inc., Easton, MD, USA) with mass spectrometry (Model LTQ XL, Thermo Fisher Scientific, Waltham, MA, USA) [18]. 

### 2.5. Analysis of Metabolic Markers and LGM

In the fasting blood sample taken prior to consumption of the MS mix solution, zonulin, LBP, and sCD14 were evaluated. Enzyme-linked immunosorbent assay (ELISA) was used to measure zonulin (K5601, Immundiagnostik AG, Bensheim, Germany) in serum and LBP (HK315-02, Hycult Biotech, Uden, The Netherlands) and sCD14 (HK320-02, Hycult Biotech) in plasma, according to the manufacturer’s protocols. Additionally, to gain a more accurate insight into the metabolic health of the subjects, the body mass index (BMI) was calculated from the heights and weights of the subjects, and the metabolic health markers glucose, LDL cholesterol, HDL cholesterol, total cholesterol, total triglycerides, ALT, GGT, and the inflammatory marker C-reactive protein (CRP) were measured in the fasting blood sample. These metabolic health markers in the blood were assessed at the hospital Gelderse Vallei (Ede, The Netherlands) using standardized clinical procedures. All blood biomarkers were assessed twice (once with and once without an acetylsalicylic acid challenge prior to the gut permeability test).

### 2.6. Statistics

SPSS statistical software version 22 (IBM Corp., Armonk, NY, USA) was used to perform the statistical analyses. Normal distribution of the markers was checked using Q–Q plots, Kolmogorov–Smirnov, and Shapiro–Wilk. An independent *t*-test was used to evaluate the significant differences between the two metabolic health groups. For all markers, values were log_10_-transformed (and ‘0’ or ‘below detection limit’ were adjusted to half of the lowest value that could be measured) to conduct Pearson’s correlation analyses. Furthermore, for LGM, only values with a coefficient of variation (CV) < 20% in the ELISA assays were considered adequate and were included in the statistical analyses (for all markers). Outliers were also included (analyzed by boxplots—IQR), as no reasonable arguments were available to exclude these samples from further analyses, and we verified that these outliers did not substantially affect the statistical outcomes, as we performed analyses with and without outliers. 

## 3. Results

### 3.1. Selection and Baseline Characteristics of Subjects

Approximately 100 individuals who responded to the study invitation met the exclusion criteria. Next, the waist circumference was measured, which was used as the first criterion to select the most metabolically healthy and metabolically unhealthy subjects. We selected 80 subjects with the highest (*n* = 40) and lowest waist circumferences (*n* = 40) for subsequent finger stick measurements to assess the fasting glucose and HDL cholesterol levels. Based on the latter values, we then selected the most metabolically healthy (*n* = 15) and metabolically unhealthy (*n* = 15) subjects, with a balance for age and sex in both groups. The mean values of the subjects in the two groups after screening are provided in Table S1. The screening parameters were significantly different (*p* < 0.05) between the groups. 

After screening, we had to exclude one subject assigned to the metabolically unhealthy group based on the waist circumference. This participant had substantial weight loss (~10 kg) due to hyperthyroidism during the period between screening and the MS permeability test. Prior to the MS permeability test (unchallenged), fasting blood samples were taken, and the metabolic health profile was assessed using standard clinical procedures. Based on these metabolic parameters in the blood, one other participant had to be excluded from the study due to exceptionally high levels of liver enzymes gamma-glutamyl transferase (GGT, >270 U/L) and alanine aminotransferase (ALT, >70 U/L), indicative of liver dysfunction. Therefore, further statistical analyses were performed with 14 subjects in the metabolically healthy group and 14 in the metabolically unhealthy group. Table 1 shows the metabolic health parameters of both the groups at baseline, prior to the MS permeability tests. The screening parameters (waist circumference, fasting glucose, and HDL cholesterol) were significantly different between both groups; however, no significant differences were found for the total cholesterol, LDL cholesterol, ALT, and CRP. (Table 1). 

### 3.2. Unchallenged MS Permeability Test

In the unchallenged MS permeability test, no significant differences in the sugar ratios that reflect the local gut permeability were found between the metabolic health groups (Table 2). Additionally, LGM, measured in the fasting blood samples prior to the MS permeability test, showed no significant differences between both groups (Table 3).

### 3.3. Challenged MS Permeability Test 

Acetylsalicylic acid is known to exacerbate (paracellular) gut permeability [20]. Therefore, a second MS permeability test was conducted with an acetylsalicylic acid pretreatment to test the resilience to a challenge that was expected to increase the difference in gut permeability between metabolically healthy and unhealthy subjects. However, no significant differences were observed between the metabolically healthy and unhealthy subjects (Table 4). Only whole gut permeability tended to be higher in the metabolically unhealthy group than in the metabolically healthy group. Prior to this MS permeability test, fasting blood was again collected to ensure that the acetylsalicylic acid pretreatment did not affect the metabolic health parameters. The fasting glucose, HDL cholesterol, triglycerides, and GGT levels were still significantly different between the metabolic health groups (Table 5), and the levels of all the parameters were highly similar to the unchallenged condition (Table 1). In these fasting blood samples, LGM was also measured, but similar to the results of the unchallenged MS permeability test, no significant differences were found for LGM between the metabolic health groups (Table 6). Furthermore, it seems that acetylsalicylic acid did not impact the levels of LGM, as they were highly similar to the unchallenged levels (Table 3). Note, however, that this study was designed as an observational study, and we must therefore be careful in making direct comparisons between the challenged (acetylsalicylic acid pretreatment) and unchallenged conditions. 

### 3.4. Correlation between Metabolic Health and Gut Permeability

**Figure 3:**
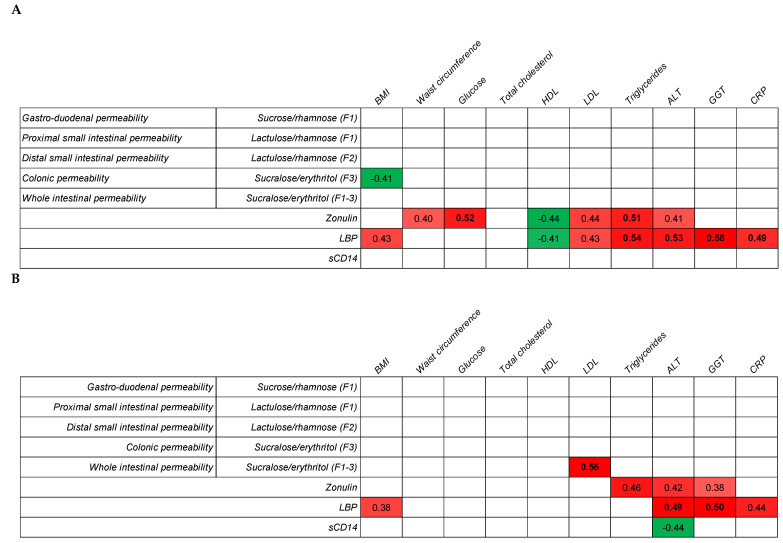
Correlation between metabolic health and gut permeability markers. Pearson’s correlation was performed on the log_10_-transformed values of metabolic health markers and sugar ratios and LGM (**A**) without an acetylsalicylic acid challenge (unchallenged) and (**B**) with an acetylsalicylic acid challenge. Nonbold: correlation is significant at the 0.05 level (2-tailed), bold: correlation is significant at the 0.01 level (2-tailed), and empty cells: no significant correlation. Red indicates a positive correlation (more intense color means a higher correlation), and green indicates a negative correlation.

Next, we analyzed the correlation between the gut integrity markers (MS permeability test and LGM) and metabolic health parameters (Figure 3). In the unchallenged condition, the LGMs zonulin and LBP showed pronounced correlations with metabolic health. Colonic permeability was negatively correlated with the BMI, while no other significant correlations were found between intestinal permeability and health biomarkers. The correlation with the individual data points in the unchallenged condition is provided in Table S2 In the acetylsalicylic acid-challenged condition, LDL cholesterol correlated with the sucralose/erythritol ratio in all three fractions (0–24 h), which reflects whole intestinal permeability. Furthermore, LGM showed a less pronounced correlation with metabolic health in the challenged condition than that in the unchallenged condition. To determine whether and how gut permeability, measured by an MS test, correlates with LGM, we performed correlation analyses for both MS permeability tests (Tables S3 and S4). However, no significant correlations were found between LGM (plasma and serum) and the urinary sugar ratio. All Pearson’s correlation analyses and anonymized datasets are provided in Tables S5 and S6.

## 4. Discussion

This study was conducted to investigate whether metabolic health could be linked to intestinal permeability, as assessed by an MS permeability test that provides site-specific information on paracellular permeability along the gastrointestinal tract. Therefore, we intended to select clearly distinctive metabolically healthy and metabolically unhealthy subjects. However, although the average BMI, fasting glucose level, and triglycerides level in the metabolically unhealthy group were significantly higher than those in the metabolically healthy group, all these subjects were still in the normal, healthy range according to the IDF global definition of metabolic syndrome [21]. This indicates that these subjects had a slightly elevated risk but not as extreme as is often seen in such studies comparing healthy individuals with diseased individuals [17,22,23].

In the unchallenged MS permeability test, no significant differences in the sugar ratios were found between the metabolically healthy and unhealthy subjects. This is in accordance with a study by Farhadi et al. [17], who found that, without an acetylsalicylic challenge, the gut permeability was not significantly different between metabolically healthy and unhealthy subjects. However, in contrast to the findings of Farhadi et al., in our study, the acetylsalicylic challenge prior to the MS permeability test did not induce substantial differences in gut permeability between metabolically healthy and unhealthy subjects. This may be explained by the fact that Farhadi et al. included patients with steatosis and NASH, i.e., those with a more pronounced disease load and high susceptibility to gut leakiness, whereas, in our study, metabolically unhealthy subjects could still be considered relatively healthy, with no known diagnosed metabolic diseases. Our power calculation was based on the study of Farhadi et al. [17], with a much more pronounced difference in metabolic health between the groups. Since, in our study, the differences between the metabolic health groups were less pronounced, we might have needed more subjects in our study to find significant differences.

The baseline intestinal permeability, as assessed by the MS permeability test, did not show convincing correlations with most of the metabolic health parameters in our study. The negative correlation between the BMI and colonic permeability was surprising and hard to explain. In the literature, mostly a positive correlation has been found, but in general, a more extreme metabolic unhealthy phenotype is included [24]. After the acetylsalicylic challenge, only LDL was correlated with the sucralose/erythritol ratio, reflecting the whole intestinal permeability. This indicates that whole gut permeability is linked to metabolic health, which is in accordance with previous studies that have reported a link between visceral fat and increased whole gut permeability [10,17]. 

In addition to the MS permeability tests, we also measured LGMs—zonulin, LBP, and sCD14—in fasting blood samples. There were no significant differences in the LGM between the two metabolic health groups, both in the unchallenged and challenged conditions. Nevertheless, the values of all LGMs seemed to be slightly higher in the metabolically unhealthy group. In our previous study [11], significant differences were detected in the LBP, sCD14, and zonulin levels between the metabolically healthy and unhealthy subjects. However, the sample size was larger (40 subjects per group instead of 14); a smaller sample size could be a reason for no significant differences in LGM in the current study. Zonulin was most consistently correlated with the metabolic health parameters, especially the waist circumference, glucose, HDL cholesterol, LDL cholesterol, triglycerides, and ALT. The link between sCD14 and metabolic health was not very robust, as none of the metabolic markers were consistently correlated with sCD14. In contrast to our study, a previous study reported that circulating sCD14 was positively associated with insulin sensitivity in morbidly obese participants, which may have a modulating effect on insulin sensitivity [25]. In regard to LBP, a consistent correlation was mostly found with the BMI, but especially strong correlations were found between triglycerides and CRP. High triglyceride levels might be linked to higher chylomicron production [26], and subsequently, chylomicrons might support LPS translocation [27]. Systemic LPS triggers higher LBP levels to elicit immune responses by presenting LPS to important cell surface receptors CD14 and TLR4 [28]. Therefore, higher LBP levels might, together with CRP, be linked with higher levels of chronic inflammation [27,29,30]. To support this potential link, further studies are needed to explore the postprandial LGM responses after a lipid challenge that will trigger fat-induced LPS translocation, such as in subjects with small intestinal bacterial overgrowth. In addition, it has been reported that microbiota profiles are different between obese and non-obese people [31]. Probiotics modulate microbiota [32] and reduce LPS in the blood [33]. This is likely the mechanism of gut barrier improvement. Thus, probiotics may be effective to prevent LPS translocation. We hypothesized that the MS permeability test would not be a good method to study differences in the gut integrity linked to metabolic health within a healthy population, as it reflects paracellular passage rather than transcellular or fatty acid transporter-facilitated translocation [27]. The absence of a significant correlation between the MS permeability test and LGMs indicates that both methods reflect a different route of intestinal permeability. Where the MS permeability test mostly reflects paracellular permeability, LGM, and especially LBP and sCD14, are likely more linked to transcellular permeability.

In the literature, there is increasing evidence that LPS is translocated over the intestinal lining via a transcellular route [27] and that this process is linked to chronic inflammation, which is a risk factor for the development of various metabolic diseases. This transcellular translocation of LPS is induced by dietary fat, and in vitro data support the link between LPS translocation, fat exposure, and chylomicron production [34]. Unfortunately, LPS is hard to measure accurately in human blood samples, and therefore, we selected LBP and sCD14 as surrogate biomarkers, as has been previously suggested in various papers [35,36,37]. LBP and sCD14 are closely linked to LPS and its activity and are also associated with obesity [38]. Furthermore, LBP and sCD14 show postprandial responses to a high fat load [39], as is also seen for LPS. Based on these data, we assume that LBP and sCD14 are accurate surrogate markers to reflect transcellular gut permeability. Zonulin is not or less expected to be directly linked to transcellular permeability. Zonulin was included in the study as it is reported to be a regulator of intestinal permeability by modifying protein–protein interactions within the tight junctions. Zonulin release is, in general, not seen as a consequence of intestinal damage (as are citrulline and I-FABP) but more as a trigger for increased gut permeability [40]. It is furthermore previously already linked to a leaky gut, chronic inflammation, and various metabolic diseases [40].

## 5. Conclusions

Taken together, we conclude that paracellular permeability, as assessed by the MS permeability test, is less correlated with metabolic health than intestinal permeability reflected by the LGMs, especially zonulin and LBP. Therefore, these LGMs are likely to be more reliable markers that reflect a ‘leaky gut’ and are associated with metabolic health.

## Figures and Tables

**Table 1:** Metabolic characteristics of metabolically healthy (*n* = 14) and metabolically unhealthy (*n* = 14) subjects at baseline.

Metabolic Parameter	Metabolically Healthy	Metabolically Unhealthy	*p*-Value *
Weight (kg)	65.7 (3.9)	88.7 (6.3)	<0.01
BMI (kg/m^2^)	21.7 (0.8)	27.9 (1.7)	<0.01
Waist circumference (cm)	81.2 (2.5)	101.1 (3.8)	<0.01
Glucose (mmol/L)	4.9 (0.1)	5.3 (0.2)	<0.01
Total cholesterol (mmol/L)	5.8 (0.4)	5.7 (0.6)	0.86
HDL (mmol/L)	1.8 (0.2)	1.3 (0.1)	<0.01
LDL (mmol/L)	3.4 (0.3)	3.6 (0.5)	0.44
Triglycerides (mmol/L)	0.8 (0.2)	1.5 (0.5)	0.02
ALT (U/L)	20.7 (3.1)	22.3 (3.0)	0.49
GGT (U/L)	13.7 (1.8)	24.7 (8.0)	0.02
CRP (mg/mL)	4.5 (1.9)	8.1 (3.3)	0.07

Data are presented as the mean (95% CI). * Significance by independent *t*-test. BMI: body mass index, ALT: alanine aminotransferase, GGT: gamma-glutamyl transpeptidase, HDL: high-density lipoprotein, LDL: low-density lipoprotein, and CRP: C-reactive protein.

**Table 2:** Urinary sugar ratios of metabolically healthy (*n* = 14) and metabolically unhealthy (*n* = 14) subjects in the unchallenged MS permeability test.

Location Permeability	Sugar Markers	Metabolically Healthy	Metabolically Unhealthy	*p*-Value *
Gastroduodenum	Sucrose/rhamnose (×10^3^) (F1)	80.3 (49.8)	55.7 (12.9)	0.36
Proximal small intestine	Lactulose/rhamnose (× 10^3^) (F1)	25.0 (12.0)	18.7 (2.5)	0.33
Distal small intestine	Lactulose/rhamnose (×10^3^) (F2)	46.4 (19.8)	45.7 (7.6)	0.95
Colon	Sucralose/erythritol (×10^3^) (F3)	17.8 (3.9)	13.8 (3.8)	0.16
Whole intestine	Sucralose/erythritol (×10^3^) (F1–3)	15.4 (3.2)	13.4 (2.2)	0.32

* Significance by independent *t*-test. F: urinary fraction.

**Table 3:** LGM levels of metabolically healthy (*n* = 14) and metabolically unhealthy (*n* = 14) subjects in the unchallenged MS permeability test.

Leaky Gut Markers (LGM)	Metabolically Healthy	Metabolically Unhealthy	*p*-Value *
Zonulin (ng/mL)	33.5 (2.9)	37.3 (4.1)	0.15
LBP (µg/mL)	12.5 (1.3)	14.2 (2.3)	0.22
sCD14 (µg/mL)	1.4 (0.1)	1.5 (0.1)	0.11

* Significance by independent *t*-test.

**Table 4:** Urinary sugar ratios of metabolically healthy (*n* = 14) and metabolically unhealthy (*n* = 14) subjects after an acetylsalicylic acid challenge.

Location Permeability	Sugar Markers	Metabolically Healthy	Metabolically Unhealthy	*p*-Value *
Gastroduodenum	Sucrose/rhamnose (×10^3^) (F1)	52.3 (17.3)	65.7 (14.5)	0.26
Proximal small intestine	Lactulose/rhamnose (×10^3^) (F1)	64.6 (14.3)	75.0 (13.0)	0.30
Distal small intestine	Lactulose/rhamnose (×10^3^) (F2)	84.8 (24.0)	81.0 (9.5)	0.77
Colon	Sucralose/erythritol (×10^3^) (F3)	15.9 (2.6)	17.2 (3.0)	0.52
Whole intestine	Sucralose/erythritol (×10^3^) (F1–3)	13.4 (1.4)	16.3 (3.0)	0.09

* Significance by independent *t*-test. F: urinary fraction.

**Table 5:** Metabolic characteristics of metabolically healthy (*n* = 14) and metabolically unhealthy (*n* = 14) subjects after an acetylsalicylic acid challenge.

Metabolic Parameter	Metabolically Healthy	Metabolically Unhealthy	*p*-Value *
Glucose (mmol/L)	4.8 (0.1)	5.2 (0.2)	<0.01
Total cholesterol (mmol/L)	5.7 (0.5)	5.3 (0.6)	0.86
HDL (mmol/L)	1.8 (0.2)	1.2 (0.1)	<0.01
LDL (mmol/L)	3.5 (0.4)	3.5 (0.5)	0.84
Triglycerides (mmol/L)	0.8 (0.2)	1.5 (0.4)	0.01
ALT (U/L)	21.3 (2.3)	22.6 (4.0)	0.58
GGT (U/L)	13.1 (1.5)	22.4 (7.0)	0.02
CRP (mg/mL)	3.7 (1.9)	7.6 (3.3)	0.06

Data are presented as the mean (95% CI). * Significance by independent *t*-test. BMI: body mass index, ALT: alanine aminotransferase, GGT: gamma-glutamyl transpeptidase, HDL: high-density lipoprotein, LDL: low-density lipoprotein, and CRP: C-reactive protein.

**Table 6:** LGM levels of metabolically healthy (*n* = 14) and metabolically unhealthy (*n* = 14) subjects after an acetylsalicylic acid challenge.

Leaky Gut Markers (LGM)	Metabolically Healthy	Metabolically Unhealthy	*p*-Value *
Zonulin (ng/mL)	31.2 (4.0)	36.3 (4.0)	0.09
LBP (µg/mL)	12.7 (1.8)	14.5 (2.2)	0.24
sCD14 (µg/mL)	1.5 (0.1)	1.6 (0.1)	0.17

* Significance by independent *t*-test.

## Data Availability

The data presented in this study are within the article and the Supplementary Materials.

## Supplementary Materials

The following are available online at https://www.mdpi.com/article/10.3390/healthcare9111583/s1, Table S1: Screening data of metabolically healthy (*n* = 15) and metabolically unhealthy (*n* = 15) subjects. Table S2: The correlations with the individual data points in the unchallenged condition. Table S3: Pearson’s correlation between LGM and the MS permeability test without an acetylsalicylic acid challenge. Table S4: Pearson’s correlation between LGM and the MS permeability test with an acetylsalicylic acid challenge. Table S5: All Pearson’s correlation analyses. Table S6: Anonymized dataset.
